# Can the Fluxionality in Borospherene Influence the Confinement-Induced Bonding between Two Noble Gas Atoms?

**DOI:** 10.3390/molecules27248683

**Published:** 2022-12-08

**Authors:** Ranita Pal, Pratim Kumar Chattaraj

**Affiliations:** 1 Advanced Technology Development Centre, Indian Institute of Technology Kharagpur, Kharagpur 721302, India; 2 Department of Chemistry, Indian Institute of Technology Kharagpur, Kharagpur 721302, India

**Keywords:** fluxionality, encapsulation, borospherene, fullerene, noble gas

## Abstract

A density functional theory study is performed to determine the stability and bonding in the neon dimer inside the B_30_N_30_ fullerene cage, the fluxional B_40_ cage, and within non-fluxional cages such as B_12_N_12_ and C_60_. The nature of bonding in the Ne_2_ encapsulated B_40_ is compared with the that in other cages in an attempt to determine whether any possible alterations are brought about by the dynamical nature of the host cage apart from the associated confinement effects. The bonding analysis includes the natural bond order (NBO), Bader’s Atoms-in-Molecules electron density analysis (AIM), and energy decomposition analysis (EDA), revealing the non-covalent nature of the interactions between the Ne atoms and that between the Ne and the cage atoms. The formation of all the Ne_2_@cage systems is thermochemically unfavourable, the least being that for the B_30_N_30_ cage, which can easily be made favourable at lower temperatures. The Ne-Ne distance is lowest in the smallest cage and increases as the cage size increase due to steric relaxation experienced by the dimer. The dynamical picture of the systems is investigated by performing ab initio molecular dynamics simulations using the atom-centred density matrix propagation (ADMP) technique, which shows the nature of the movement of the dimer inside the cages, and by the fact that since it moves as a single entity, a weak bonding force holds them together, apart from their proven kinetic stability.

## 1. Introduction

Encapsulation of noble gas (Ng) atoms and their dimers is widely studied in many caged systems in an attempt to understand the possible bonding between the so-called “inert” elements. Owing to their high and low IP and EA, respectively, noble gases tend to be chemically “inert: towards other elements unless they are subjected to a strong polarizing source that would facilitate a deformation in their otherwise rigid electron density to induce a donor–acceptor type of interaction. Noble gas dimers only have weak dispersive interactions within them. Confining them within molecular cages allows them to interact with the cage atoms as well as with each other. Although in most cases the complex formation is not thermochemically favourable, they have kinetic stability and hence do not disintegrate easily once formed. The most common caged compounds to form stable endohedral complexes with varied types of atoms and molecules are the carbon fullerene compounds. Confining noble gas atoms within neutral as well as cationic fullerene systems was experimentally performed using high-energy bimolecular collision reactions for the charged ones. For the neutral systems, generating C_60_ from graphite in a He atmosphere, using high temperatures to achieve the “window mechanism” where the Ng atoms are inserted via the formation of a temporary window by breaking one or more of the cage C-C bonds, was adopted. [1,2,3,4,5,6,7,8,9,10,11,12,13,14]. Later on, the insertion process involved the use of cationic Ng beams on the fullerene cage [15], “molecular surgery” [16,17], etc. The study of endohedral metallofullerenes is also being explored in recent times [18]. Guest–host confined systems with a single Ng atom (He-X) [14] and with Ng dimers within the cavity of C_60_ have been reported both experimentally [19] as well as theoretically, along with that of several other small molecules such as HF, CO_2_, H_2_, H_2_O, etc. [20,21,22,23,24]. Soon after the theoretical prediction of noble gas dimers trapped within fullerene [25], the experimental detection of He_2_@C_70_ and Ne_2_@C_70_ was reported through NMR and mass spectrometry [26,27]. The confined Ng atoms within the fullerene cages exhibit a van der Waals type of interaction between them to attain stability in most cases. There exists no genuine chemical bond between the guest atoms and the cage C atoms. However, the presence of a “chemical bond” between two Xe atoms confined with the C_60_ cage is claimed by Krapp and Frenking [20]. Other than fullerenes, noble gas confinement within cages with much smaller cavities such as C_10_H_16_, C_20_H_20_ and Mo_6_Cl_8_F_6_ [28,29,30,31], along with those within smaller BN-fullerenes, such as B_12_N_12_ and B_16_N_16_, [32] is also performed to study their viability and stability.

Pure boron cages are also explored in this regard. Among them, the borospherene, B_40_ cage, shows some interesting features regarding its dynamical behaviour. It contains four numbers of heptagonal and two hexagonal holes, with a HOMO-LUMO energy gap comparable to that of C_60_. A molecular dynamics study on the molecule reveals the dynamical behaviour of the cluster arising from a continuous interconversion between the two types of holes (B_6_ and B_7_) [33]. The B_40_ molecule has been previously used to trap Ng atoms, and the dynamical behaviour of the Ng@B_40_ system is similar to that of the bare cage [34]. The C_60_, on the other hand, cannot undergo rapid interconversion among its isomers due to its rigid σ-framework. It contains 12 5-C and 20 6-C rings [35]. Previously, it was proposed that any B, N doped structures of fullerene should not have any B-B or N-N bonds. This required the B, N doped structure of C_60_ to have one C atom in all the five-membered rings, thus producing C_12_B_24_N_24_ [36]. However, studies on the B_30_N_30_ ring show that it forms a stable structure, and it was also hypothesized that it can be easily synthesized from borazine [37]. We have encapsulated neon dimers within this cage to study the stability of the resulting complex and the bonding situation therein. The nature of bonding in the noble-gas-encapsulated borospherene is also investigated and compared with those of B_12_N_12_, B_30_N_30,_ and C_60_ cages in an attempt to determine whether any possible alterations are brought about by the dynamical nature of the host cage apart from that arising out of confinement. Density functional theory-based computations are performed, and the bonding analysis includes the natural bond order (NBO) [38,39] Bader’s Atoms-in-Molecules electron density analysis (AIM) [40], and energy decomposition analysis (EDA) [41,42,43]. The dynamical picture of the systems is investigated by performing ab initio molecular dynamics simulations using the atom-centred density matrix propagation (ADMP) [44,45,46] technique.

## 2. Methods and Computational Details

Geometry optimizations and frequency calculations for the B_40_, B_12_N_12_, Ne_2_@B_40,_ and Ne_2_@B_12_N_12_ systems are performed using the DFT functional, ωB97X-D [47] with 6-311G(d,p) [48,49] basis set, while those of the larger systems, B_30_N_30_, C_60_, Ne_2_@B_30_N_30,_ and Ne_2_@C_60_, are performed using the 6-31G [50,51] basis set with the same DFT functional. The dissociation energies calculated for the system use the following equation:

(1) $$D_{e}=E_{Ne_{2}}+E_{cage}-E_{Ne_{2}@cage}$$

The energy corresponding to the distortion that the host undergoes due to the encapsulation of the guest molecules (*E*_dist_) is calculated as follows:

(2) $$E_{\mathrm{dist}}=E_{expanded\;cage}-E_{cage}$$

where *E*_dist_ is obtained by removing the Ne_2_ molecule and by evaluating single-point energy of the expanded cage. Basis set superposition error (BSSE) is corrected by using the counterpoise method [52].

The natural charge on the atoms and Wiberg bond index between two atoms are calculated using NBO 3.1 [38] at the ωB97X-D/6-311G (d,p) level of theory. Electron density analysis is performed using the Multiwfn software [53] to calculate the relevant topological descriptors. The energy decomposition analysis performed at B3LYP-D3(BJ)/TZ2P//ωB97X-D/6-311G(d,p) (for Ne_2_@B_40_ and Ne_2_@B_12_N_12_) and B3LYP-D3(BJ)/TZ2P//ωB97X-D/6-31G (for Ne_2_@B_30_N_30_ and Ne_2_@C_60_) levels uses the ADF 2014.01 software [54,55] to evaluate different types of interactions between the Ne_2_ and the respective cages. The interaction energy between two relevant fragments is evaluated as the total of the Pauli repulsive interaction (Δ*E*_Pauli_) and three attractive interaction energies, viz., electrostatic (Δ*E*_elstat_,), orbital (Δ*E*_orb_), and dispersive (Δ*E*_disp_) interactions. Lastly, atom-centered density matrix propagation (ADMP) is utilized to carry out the ab initio molecular dynamics simulation. All the above computations are executed using the Gaussian 16 [56] program package in the supercomputing facility, PARAM Shakti, at IIT Kharagpur.

## 3. Results and Discussion

### 3.1. Structure and Stability

**Figure 1:**
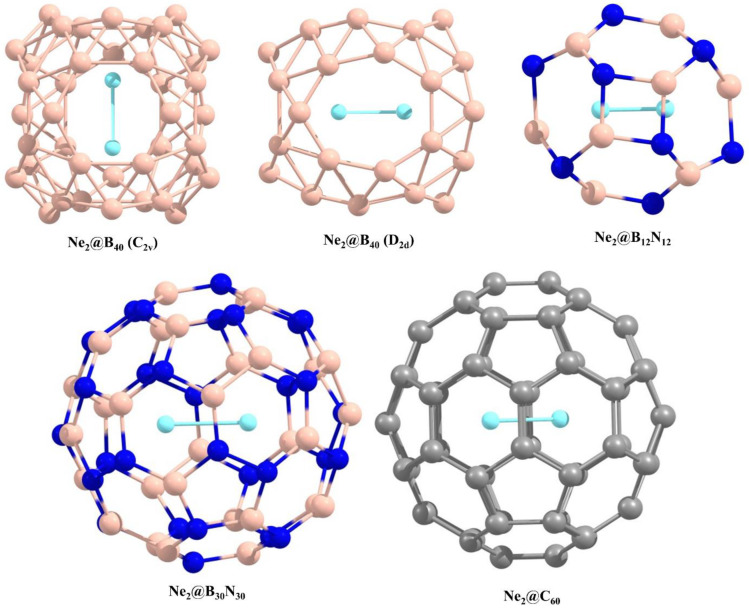
Optimized geometries of the Ne_2_-encapsulated cages. The boron, carbon, nitrogen, and neon atoms are indicated by light pink, grey, dark blue, and sky blue colours, respectively.

The cages considered for encapsulating neon dimers are the fluxional B_40_, the non-fluxional B_12_N_12_, and the fullerenes C_60_ and B_30_N_30_. The optimized geometries of the encapsulated cage systems are provided in Figure 1. The fluxional borospherene cage has a *D*_2*d*_ symmetry with an ^1^*A*_1_ electronic state. The B_40_ cage has two six-membered and four seven-membered ring structures, the continuous interconversion among which causes its fluxional behaviour akin to that of a nanobubble. On encapsulation of Ne_2_, two possible geometries can be obtained, one with a *C*_2*v*_ symmetry and the other with a *D*_2*d*_ symmetry, where the former is marginally more stable. The bond axis of Ne_2_ is oriented along the imaginary line connecting the midpoints of the two opposing B_7_ rings in the former case and the B_6_ rings in the latter case. The formation of both of these Ne_2_@B_40_ systems is thermochemically unfavourable (see Table 1); however, once formed, they have kinetic activation barriers high enough to protect from their spontaneous dissociation (~67.5 kcal mol^−1^ at ωB97X-D/def2-TZVP//ωB97X-D/def2-SVP level) [34]. The distance between the Ne atoms within the cavity reduces from 3.061 to 1.913 Å and to 1.877 Å in the *C*_2*v*_ and *D*_2*d*_ isomers, respectively. The distortion undergone by the host cage as a result of confining the Ne dimers within it is calculated as 6.395 and 8.106 kcal mol^−1^ for the *C*_2*v*_ and *D*_2*d*_ isomers, respectively.

The B_30_N_30_ cage is a BN analogue of the C_60_ fullerene. Out of the three possible geometries, as reported by Yin et al. [57], we have selected the one with the minimum energy where the cage contains six N-N bonds. It primarily differs from carbon fullerene in the fact that, unlike C_60_, it has non-uniform diameters of the rings throughout the cage and is slightly larger than C_60_. The encapsulation of Ne_2_ within this cage is marginally unfavourable at room temperature (Δ*G* for the dissociation process is −5.073 kcal mol^−1^), which indicates that at slightly lower temperatures, its formation will be thermochemically feasible. The interatomic distance between the Ne atoms is found to be 2.130 Å, which is relatively larger than that in the B_40_ cage owing to the availability of ample space within the B_30_N_30_ cage. For this reason, the encapsulation of dimers of higher noble gas atoms is also viable without distorting the cage, much like the case in its carbon analogue. The B_12_N_12_ cage, on the other hand, has a much smaller cavity with four- and six-membered rings. On Ne_2_ encapsulation, it becomes distorted to a much higher extent than all the other cages in consideration in this study (see Table 1). The Ne-Ne distance is also much lower (1.602 Å) due to the higher confinement effect of the small host cage. All attempts of inserting Ar_2_ within the cage failed due to insufficient space within the cage. Insertion of Ng dimers within C_60_ has been reported earlier. It can accommodate dimers of higher noble gas elements as well with distortions increasing with increasing size of the Ng atoms. In the case of Ne_2_@C_60_, the internuclear distance between the Ne atoms is 2.050 Å. Several geometries with different point group symmetries are possible for this system, which are energetically close to each other. This occurs due to the precessional motion of the dimer inside the cage [58].

Table 1 presents the zero-point corrected dissociation energy (ZPE, *D*_0_), both ZPE and BSSE corrected dissociation energy (*D*_0_^BSSE^), entropy change (Δ*S*) and free energy change (Δ*G*) for the dissociation processes, Ne_2_@cage → Ne_2_ + cage, and Ne_2_@cage → Ne + Ne@cage. The *D*_0_^BSSE^ has higher negative values than the corresponding *D*_0_ values. From the distortion and dissociation energy values, it is clear that the amount of destabilization to the cage caused by the Ne_2_ encapsulation decreases with their increasing cavity size. Although the Ne_2_@B_30_N_30_ cage showed a small positive dissociation energy, the introduction of the BSSE correction produced a small negative dissociation energy. Among all the cages considered, the encapsulation of Ne_2_ within the B_30_N_30_ cage seems to be the most likely to be made favourable at lower temperatures. To determine the reaction energy of the process, 2Ne + cage → [Ne_2_@cage] (where the third brackets indicate that the system is frozen in that geometry), we need to calculate the energies step by step: first, the energy required to bring the two Ne atoms to their equilibrium distance within the cages, i.e., 2Ne → [Ne_2_], where [Ne_2_] indicates the frozen geometry of Ne_2_ as obtained within the respective cages; second, the energy [*E*_dist_] involved in the distortion of the cages in their equilibrium state to the geometry they attain after encapsulating the Ne_2_, i.e., cage → [cage]; finally, the interaction energy between the frozen dimer and cage, i.e., [Ne_2_] + [cage] → [Ne_2_@cage]. The aforementioned energies are tabulated in Table 2.

### 3.2. Bonding

The interactions between the two Ne atoms inside the cages are investigated in terms of NBO, AIM, and EDA. The internuclear bond distances of the free Ne dimer is reported to be 3.091 Å [59], and the atoms are bound to each other with weak van der Waals forces of attraction. In the studied complexes, the Ne-Ne distances are 1.913 and 1.877 Å in the *C*_2*v*_ and *D*_2*d*_ isomers of Ne_2_@B_40_, and 1.602, 2.130, and 2.050 Å in Ne_2_@B_12_N_12_, Ne_2_@B_30_N_30_, and Ne_2_@C_60_ systems, respectively. There is clearly an increase in the interatomic interaction, the extent of which is discussed to determine whether the resulting situation can be considered a genuine chemical bond.

The natural charges on the Ne atoms calculated within the cage systems are much higher in the smaller B_12_N_12_ (*q*_Ne_ = 0.137 |e|) than in the larger cages (*q*_Ne_ = 0.047 |e|, 0.042 |e|, and 0.010 |e| in B_40_, B_30_N_30_, and C_60_, respectively) (Table 3). The charge on the cage atoms of the bare host in B_12_N_12_ is 1.164 |e| (positive on the B and negative on the N atoms, respectively), which increases after encapsulating the Ne_2_. *q*_B_ and *q*_N_ ranges within 1.221–1.301 |e| and −1.252–−1.272 |e|, respectively, in the Ne_2_@B_12_N_12_ system. The B atoms in the fluxional B_40_ cage have varied charges. In each of the two B_6_ rings, two opposing B atoms have *q*_B_ = −0.210 |e| while the others have *q*_B_ = 0.118 |e|, whereas the charges on the rest of the B atoms constituting the B_7_ rings range within −0.035–0.121 |e|. In the case of B_30_N_30_ and C_60_, very little to no change is observed in the natural charges on the cage atoms. Positive charges on the Ne atoms on encapsulation indicate that a partial charge cloud transfer occurs from them to the caged atoms. Wiberg bond indices calculated for the interaction between the two Ne atoms reveal very low values (ranging within 0.0003−0.0004), which are just marginally higher than that in the free Ne dimer (0.0002). WBI values in such low ranges reveal the presence of a van der Waals type of interaction and the absence of any covalent interactions. The total WBI values for the Ne atoms are 0.274, 0.102 (and 0.100), 0.0837, and 0.0231 in Ne_2_@B_12_N_12_, *C*_2*v*_ (and *D*_2*d*_) isomer of Ne_2_@B_40_, Ne_2_@B_30_N_30_, and Ne_2_@C_60_, respectively. The higher the total WBI values, the higher the extent of interactions between the Ne and the respective cage atoms.

**Figure 2:**
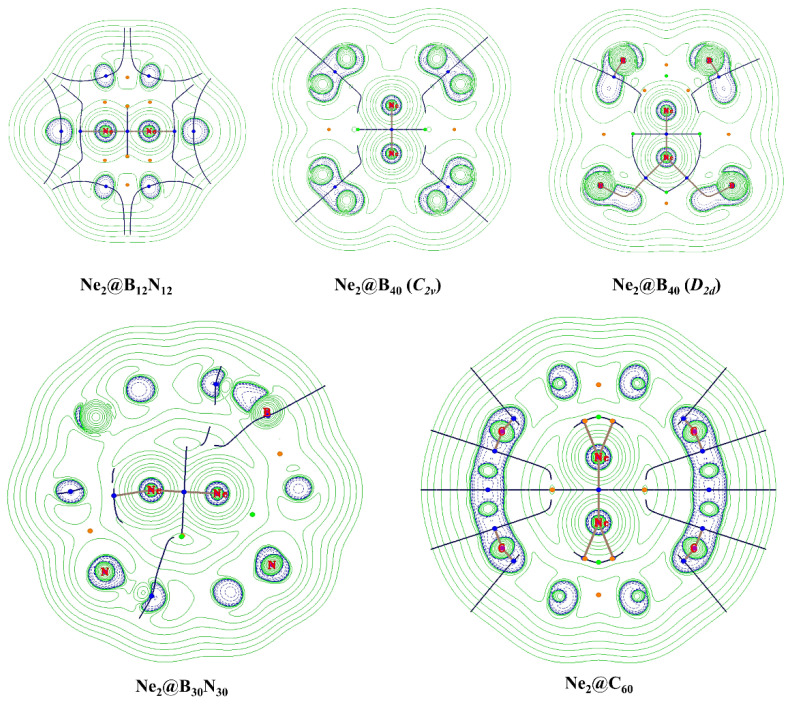
Contour diagrams of the Laplacian of the electron density of the Ne_2_@cage systems. Green solid and blue dashed lines stand for positive and negative Laplacian, respectively.

**Figure 3:**
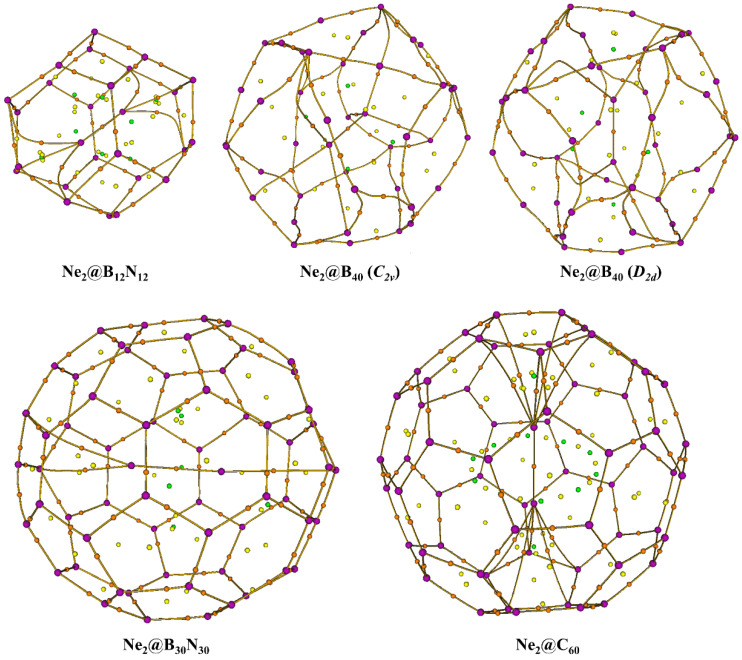
Molecular graphs generated for the Ne_2_@cage systems showing the bond paths.

Bader once claimed that the presence of a bond path between two atoms in a molecule in its equilibrium state indicates that there exists a bond between the two [60]. This statement is quite controversial [61,62,63,64,65,66] and is proven not to be true in many cases. We, therefore, rely on the AIM topological analysis to determine the nature of the interaction, rather than claim the presence of a bond just based on the presence of a bond path. Figure 2 depicts the contour diagrams for the Laplacian of the electron density, ∇^2^*ρ*(*r*_c_), calculated for the Ne_2_@cage systems. Ne-Ne bond paths are present in all the systems. Six numbers of Ne-N bond paths are detected in the Ne_2_@B_12_N_12_ system, three Ne-N in Ne_2_@B_30_N_30_, six and four Ne-B in the *C*_2v_ and *D*_2d_ isomers of Ne_2_@B_40_, respectively, and ten Ne-C in Ne_2_@C_60_ (see Figure 3). We are interested in the nature of interactions that exists between the two Ne atoms under confinement; thus, we have calculated the relevant topological descriptors, viz., electron density (*ρ*(*r*_c_)), its Laplacian (∇^2^*ρ*(*r*_c_)), local potential, kinetic, and total energy densities (*V*(*r*_c_), *G*(*r*_c_), and *H*(*r*_c_)), at the bond critical point (BCP) between the said atoms (Table 4). A positive value of the Laplacian of electron density indicates a depletion of the same, suggesting a noncovalent type of interaction. Although it is a necessary criterion, it is not a sufficient one. A positive value of the summation of the local energy densities (i.e., *H*(*r*_c_) = *G*(*r*_c_) + *V*(*r*_c_)) along with positive ∇^2^*ρ*(*r*_c_) again points towards the nature of interaction being a non-covalent one [67]. In our cases, the *H*(*r*_c_) values are positive, but their values are so small (pretty close to zero) that they may change their sign in case we try different levels of theory and stroke all basis sets. It may be noted that for the dimers of larger Ng atoms such as Kr_2_ and Ar_2_, negative *H*(*r*_c_) values are obtained [34]. This means that a partial covalent bond exists in those two dimers. It follows the prognosis made by Linus Pauling that it is possible for heavier Ng atoms to form bonds owing to the fact that they contain loosely bound electrons [68]. The magnitude of *ρ*(*r*_c_), ∇^2^*ρ*(*r*_c_), *G*(*r*_c_) and *V*(*r*_c_) gradually decreases with the increase in the size of the cage, whereas *H*(*r*_c_) increases. The ratio −*G*(*r*_c_)/*V*(*r*_c_) > 1 in all the systems suggests a purely non-covalent interaction between the Ne atoms [69]. Again, the ratio of *G*(*r*_c_) to *ρ*(*r*_c_) provides an insight into the type of interaction: greater than 1 suggests the absence of any covalent interactions. It has earlier been reported that in the B_12_N_12_ cage, some degree of covalent character can be induced between two He atoms [32,70]. Thus, this interesting behaviour of developing partial covalent bonding interaction can be investigated for heavier noble gas dimers as well by subjecting them to a similar degree of confinement. Of course, that would require increasing size of cages for increasing size of the noble gas elements.

The energy decomposition analysis is carried out considering Ne_2_ and the cage as two separate fragments. The total interaction energies between the Ne dimer and the different cages are provided in Table 5 along with its components. The Pauli interaction energy is always positive, being repulsive in nature, and it decreases with increasing size of the host cage, highest and lowest corresponding to B_12_N_12_ and C_60_, respectively. Among the attractive type of interaction energies, the electrostatic interaction dominates the other two. The percentage contribution to the net attractive energy of Δ*E*_elstat_, Δ*E*_orb_, and Δ*E*_disp_ are provided in parentheses, which clearly supports the above statement. In all the cases, the repulsive interaction is so high that it overcompensates all the attractive energies to result in an overall repulsive (positive) total interaction energy, the extent of which decreases with increasing cage size. Substantial orbital contribution is present in the case of Ne_2_@B_12_N_12_, which decreases in the larger cages, whereas that of the dispersion interaction increases with cage size.

### 3.3. ADMP

The interconversion between the six- and seven-membered ring structures in the borospherene cage causes its fluxional behaviour, which has an activation free energy barrier of 14.3 kcal mol^−1^ [33]. Such transformation is possible owing to the appropriate *σ*- and *π*- delocalization among the two possible isomers along with the corresponding transition state. The above transformation is observed in BOMD simulations performed at high temperatures of 1200 and 1500 K, which occur by the movement of one B atom during cage distortion from one of the heptagonal to the neighbouring hexagonal ring. Note that the higher temperatures are not indicative of the fact that the transformation occurs at those temperatures; this means that the higher temperatures help the system overcome the energy barrier within the very small simulation time window (in the order of *ps*). In realistic time scales, this can be observed at far lower temperatures. Upon encapsulating the noble gas monomer, no significant change is observed in the dynamical behaviour of the cage. ADMP simulations performed for the *C*_2*v*_ isomer of the dimer-encapsulated B_40_ at 400 K for a runtime of 500 fs show that the dimer undergoes slight precessional motion along the axis joining the centres of the two opposing heptagonal rings. The interconversions between the B_6_ and B_7_ rings are also observed (see simulation Video S1). Due to the smaller cavity size in B_12_N_12_, such movement of the guest molecule is restricted. Despite the larger sizes of the B_30_N_30_ and C_60_ cages, the Ne_2_ inside their cavities does not move separately, but as a single entity, proving the existence of some bonding interaction, *albeit* weak, that holds them together. The dynamical study of the empty C_60_ cage investigated earlier at higher temperatures reported no fluxional behaviour because of the fact that the associated energy barrier for the transformation to its other isomeric forms is very high and is thermally forbidden by Woodward–Hoffmann rules [71].

## 4. Conclusions

The fluxional behaviour in any system arises due to the presence of low-lying energetically accessible isomers on the potential energy surface. If the energy barrier is low enough, the interconversion is easily observed. In the case of caged systems, this phenomenon can be monitored and utilized to influence the bonding, stability, and reactivity of any confined system or to catalyse a chemical reaction. The stability and bonding in the neon dimer are studied inside the fluxional B_40_ and some non-fluxional cages such as B_12_N_12_, B_30_N_30_, and C_60_ using a density functional theory approach. The formations of all the Ne_2_@cage systems are thermochemically unfavourable, the least being that for the B_30_N_30_ cage, which can easily be made favourable at lower temperatures. The Ne-Ne distance is lowest in the smallest cage, and it increases as the cage size increases due to steric relaxation experienced by the dimer. NBO, AIM, and EDA reveal the non-covalent nature of the interaction between the Ne atoms and that between the Ne and the cage atoms. The dynamical study revealed the nature of movement of the dimer inside the cages and the fact that since it moves as a single entity, a weak bonding force holds them together. Since the fluxionality of the borospherene cage exists at a relatively higher temperature range, its effect on the bonding aspects is not very pronounced at lower temperatures. Additionally, the cage is too small to effectively host a chemical reaction without rupturing. Larger-sized fluxional cages may be inspected with different common reactions within to determine if a dynamic coupling exists between the confined reaction and the fluxional conversion (if so, whether both of them happen simultaneously or sequentially). The combined effect of confinement and fluxionality on certain chemical reactions may lead to some interesting and unusual results.

## Figures and Tables

**Table 1:** Zero-point (ZPE) corrected dissociation energy (*D*_0_), both ZPE and BSSE corrected dissociation energy (*D*_0_^BSSE^), entropy change (Δ*S*) and free energy change (Δ*G*) for the dissociation processes, Ne_2_@cage → Ne_2_ + cage, and Ne_2_@cage → Ne + Ne@cage along with the distortion energy (*E*_dist_) calculated as Equation (2). The energy values are provided in kcal mol^−1^ and the entropy change in kcal mol^−1^ K^−1^.

Systems	*E*_dist_	Processes	*D*_0_	*D*_0_^BSSE^	Δ*S*	Δ*G*
Ne_2_@B_40_ (*C*_2*v*_)	6.395	Ne_2_@B_40_ → Ne_2_ + B_40_	−70.5	−81.5	0.048	−83.5
		Ne_2_@B_40_ → Ne + Ne@B_40_	−69.3	−80.3	0.030	−77.4
Ne_2_@B_40_ (*D*_2*d*_)	8.106	Ne_2_@B_40_ → Ne_2_ + B_40_	−79.4	−90.6	0.049	−92.7
		Ne_2_@B_40_ → Ne + Ne@B_40_	−78.1	−89.4	0.031	−86.5
Ne_2_@B_12_N_12_	84.687	Ne_2_@B_12_N_12_ → Ne_2_ + B_12_N_12_	−449.8	−462.2	0.047	−462.7
		Ne_2_@B_12_N_12_ → Ne + Ne@B_12_N_12_	−352.1	−364.5	0.026	−359.7
Ne_2_@B_30_N_30_	0.604	Ne_2_@B_30_N_30_ → Ne_2_ + B_30_N_30_	5.3	−11.4	0.035	−5.1
		Ne_2_@B_30_N_30_ → Ne + Ne@B_30_N_30_	−6.7	−23.5	0.030	−15.1
Ne_2_@C_60_	0.983	Ne_2_@C_60_ → Ne_2_ + C_60_	−6.9	−19.4	0.040	−31.0
		Ne_2_@C_60_ → Ne + Ne@C_60_	−13.5	−26.0	0.033	−35.1

**Table 2:** Reaction energies calculated for all Ne_2_@cage systems.

Processes	Reactions	*E*_R_ (kcal mol^−1^)
2Ne → [Ne_2_]	$2\mathrm{Ne}\;\to \;{\left[{\mathrm{Ne}}_{2}\right]}_{\mathrm{in}\;\left[{\mathrm{Ne}}_{2}@B_{40}\right]\;\left(C_{2v}\right)}$	12.3
	$2\mathrm{Ne}\;\to \;{\left[{\mathrm{Ne}}_{2}\right]}_{\mathrm{in}\;\left[{\mathrm{Ne}}_{2}@B_{40}\right]\;\left(D_{2d}\right)}$	14.7
	$2\mathrm{Ne}\;\to \;{\left[{\mathrm{Ne}}_{2}\right]}_{\mathrm{in}\;\left[{\mathrm{Ne}}_{2}@B_{12}N_{12}\right]\;}$	54.8
	$2\mathrm{Ne}\;\to \;{\left[{\mathrm{Ne}}_{2}\right]}_{\mathrm{in}\;\left[{\mathrm{Ne}}_{2}@B_{30}N_{30}\right]\;}$	3.7
	$2\mathrm{Ne}\;\to \;{\left[{\mathrm{Ne}}_{2}\right]}_{\mathrm{in}\;\left[{\mathrm{Ne}}_{2}@C_{60}\right]}$	5.8
cage → [cage]	$B_{40}\;\to \;{\left[B_{40}\right]}_{\mathrm{in}\;\left[{\mathrm{Ne}}_{2}@B_{40}\right]\;\left(C_{2v}\right)}$	6.4
	$B_{40}\;\to \;{\left[B_{40}\right]}_{\mathrm{in}\;\left[{\mathrm{Ne}}_{2}@B_{40}\right]\;\left(D_{2d}\right)}$	8.1
	$B_{12}N_{12}\;\to \;{\left[B_{12}N_{12}\right]}_{\mathrm{in}\;\left[{\mathrm{Ne}}_{2}@B_{12}N_{12}\right]\;}$	84.7
	$B_{30}N_{30}\;\to \;{\left[B_{30}N_{30}\right]}_{\mathrm{in}\;\left[{\mathrm{Ne}}_{2}@B_{30}N_{30}\right]\;}$	0.6
	$C_{60}\;\to \;{\left[C_{60}\right]}_{\mathrm{in}\;\left[{\mathrm{Ne}}_{2}@C_{60}\right]}$	1.0
[Ne_2_] + [cage] → [Ne_2_@cage]	[Ne_2_] + [B_40_] → [Ne_2_@B_40_] (*C*_2*v*_)	50.3
	[Ne_2_] + [B_40_] → [Ne_2_@B_40_] (*D*_2*d*_)	55.1
	[Ne_2_] + [B_12_N_12_] → [Ne_2_@B_12_N_12_]	312.8
	[Ne_2_] + [B_30_N_30_] → [Ne_2_@B_30_N_30_]	−10.9
	[Ne_2_] + [C_60_] → [Ne_2_@C_60_]	11.5
2Ne + cage → [Ne_2_@cage]	2Ne + B_40_ → [Ne_2_@B_40_] (*C*_2*v*_)	69.0
	2Ne + B_40_ → [Ne_2_@B_40_] (*D*_2*d*_)	77.8
	2Ne + B_12_N_12_ → [Ne_2_@B_12_N_12_]	452.2
	2Ne + B_30_N_30_ → [Ne_2_@B_30_N_30_]	−6.6
	2Ne + C_60_ → [Ne_2_@C_60_]	18.3

**Table 3:** Natural charges on the encapsulated Ne atoms, the WBI values between two Ne atoms, and the total WBI value of Ne atoms.

Systems	*q*_Ne(1)_	*q*_Ne(2)_	WBI_Ne-Ne_	Total WBI_Ne_
Ne_2_@B_12_N_12_	0.137	0.137	0.0027	0.2736
Ne_2_@B_40_ (*C*_2*v*_)	0.047	0.047	0.0003	0.1015
Ne_2_@B_40_ (*D*_2*d*_)	0.046	0.046	0.0004	0.1001
Ne_2_@C_60_	0.010	0.010	0.0251	0.0231
Ne_2_@B_30_N_30_	0.041	0.042	0.0003	0.0837

**Table 4:** Topological descriptors (a.u.) at the bond critical points (BCPs) between Ng atoms in Ne_2_@cage systems.

Systems	*ρ*	∇^2^*ρ*(*r*_c_)	*G*(*r_c_*)	*V*(*r_c_*)	−*G*(*r*_c_)/*V*(*r*_c_)	*H*(*r_c_*)	*G*(*r_c_*)/*ρ*(*r_c_*)	ELF
Ne_2_@B_12_N_12_	0.130	1.255	0.313	−0.312	1.002	0.001	2.412	0.085
Ne_2_@B_40_ (C_2v_)	0.053	0.446	0.103	−0.095	1.089	0.008	1.933	0.042
Ne_2_@B_40_ (D_2d_)	0.059	0.497	0.116	−0.108	1.074	0.008	1.974	0.046
Ne_2_@C_60_	0.036	0.298	0.064	−0.054	1.184	0.010	1.810	0.029
Ne_2_@B_30_N_30_	0.029	0.234	0.049	−0.040	1.237	0.009	1.716	0.024

**Table 5:** Total interaction energy values and components in kcal mol^−1^ obtained from EDA results Ne_2_@cage systems using Ne_2_ and the cage as the fragments.

Systems	Δ*E*_int_	Δ*E*_Pauli_	Δ*E*_elstat_	Δ*E*_orb_	Δ*E*_disp_
Ne_2_@B_12_N_12_	326.0	666.0	−249.4 (73.4)	−82.9 (24.4)	−7.7 (2.3)
Ne_2_@B_40_ (*C*_2v_)	59.0	132.9	−51.0 (69.0)	−11.7 (15.8)	−11.2 (15.2)
Ne_2_@B_40_ (*D*_2d_)	64.0	142.4	−54.5 (69.4)	−12.7 (16.2)	−11.3 (14.3)
Ne_2_@C_60_	7.6	35.0	−14.2 (51.6)	−2.2 (8.0)	−11.1 (40.4)
Ne_2_@B_30_N_30_	4.2	28.1	−11.6 (48.4)	−2.0 (8.2)	−10.4 (43.4)

## Data Availability

Not applicable.

## Supplementary Materials

The following supporting information can be downloaded at: https://www.mdpi.com/article/10.3390/molecules27248683/s1.

## Sample Availability

Cartesian coordinates for the optimized geometries of the compounds are available from the authors on reasonable request.
